# Supplementary material for: Calreticulin Regulates SARS-CoV-2 Spike Protein Turnover and Modulates SARS-CoV-2 Infectivity
Source: Cells. 2023 Nov 23;12(23):2694. doi: 10.3390/cells12232694 (PMC10705507; doi:10.3390/cells12232694)
Supplement: Supplementary file 1 [file cells-12-02694-s001.zip › cells-2709899-supplementary.pdf]

cells

MDPI

Article

Supplemental Data

Calreticulin Regulates SARS-CoV-2 Spike Protein Turnover and Modulates SARS-CoV-2 Infectivity

Nader Rahimi [1,*], Mitchell R. White [2,3], Razie Amraei [1], Saran Lotfollahzadeh [4], Chaoshuang Xia [5], Marek Michalak [6], Catherine E. Costello [5] and Elke Mühlberger [2,3]

1 Department of Pathology, School of Medicine, Boston University, Boston, MA 02118, USA; ramraei@bu.edu
2 Department of Microbiology, School of Medicine, Boston University, Boston, MA 02118, USA; mitchw@bu.edu (M.R.W.); muehlber@bu.edu (E.M.)
3 National Emerging Infectious Diseases Laboratories (NEIDL), Boston University, Boston, MA 02118, USA
4 Renal Section, Department of Medicine, Medical Center, Boston University, Boston, MA 02118, USA; slotfoll@bu.edu
5 Center for Biomedical Mass Spectrometry, School of Medicine, Boston University, Boston, MA 02118, USA; csxia@bu.edu (C.X.); cecmsms@bu.edu (C.E.C.)
6 Department of Biochemistry, University of Alberta, Edmonton, AB T6G 2H7, Canada; mmichala@ualberta.ca
* Correspondence: nrahimi@bu.edu

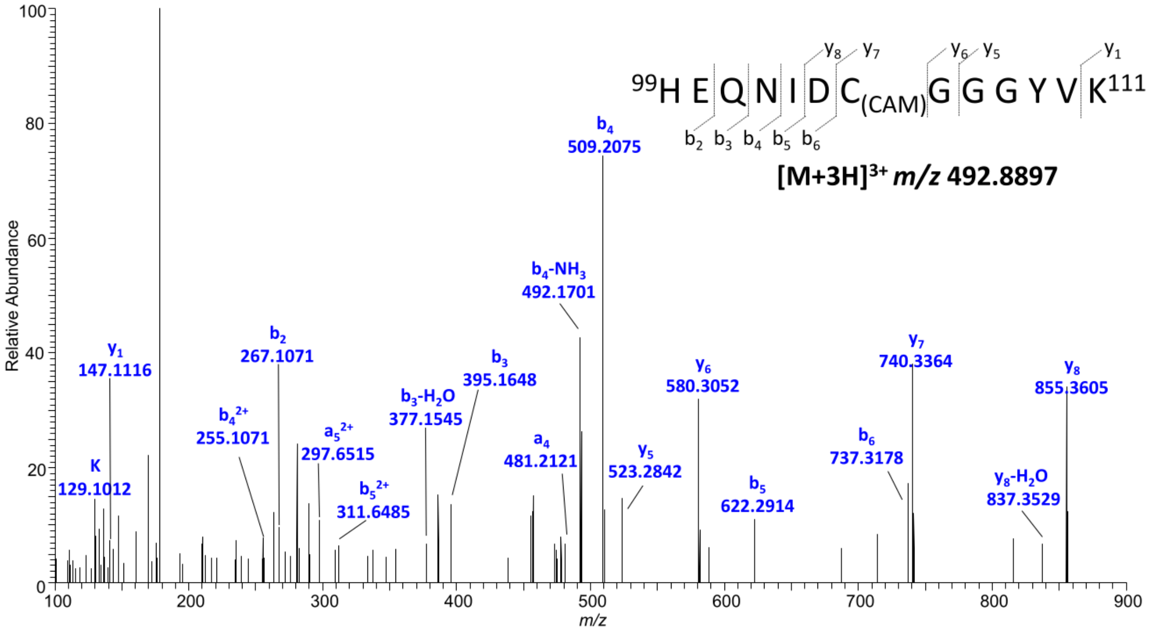

Figure S1. Whole cell lysates from HUVEC-TERT cells were incubated with S-RBD-HIS-STRIP-Ni-IMAG beads or Ni-IMAG beads alone, and the captured proteins were resolved on SDS-PAGE and stained with Coomassie Blue. The protein bands were cut out, subjected to in-gel digestion with trypsin, and the resulting peptide mixture was analyzed by LC-MS/MS. Higher energy Collision-induced Dissociation (HCD) MS/MS spectrum is assigned to the CALR peptide, ^{99}HEQNIDCGGGYVK^{111} precursor [M + 3H]3+ calc. m/z 492.8897, obs. m/z 492.8894. Symbols: b_n = N-terminal fragments; y_n = C-terminal fragments.
